# Thixotropic behaviour of thickened sewage sludge

**DOI:** 10.1186/2052-336X-12-72

**Published:** 2014-04-23

**Authors:** Petr Trávníček, Petr Junga

**Affiliations:** 1 Department of Agricultural, Food and Environmental Engineering, Mendel University in Brno, Zemedelska 1, 613 00 Brno, Czech Republic

**Keywords:** Rheology, Mathematical model, Herschel-Bulkley, Sewage sludge, Pressure flotation

## Abstract

The aim of the work is a description of the rheological behaviour of thickened sewage sludge. The sample of thickened sludge was collected from the wastewater treatment plant, where pressure flotation unit is used for a process of thickening. The value of dry matter of collected sample was 3.52%. Subsequently the sample was diluted and the rheological properties of individual samples were obtained. Several types of rheological tests were used for the determination of the sample. At first the hysteresis loop test was performed. The next test was focused on the time-dependency, i.e. measurement of dependence of dynamic viscosity on the time at constant shear rate. Further dependence dynamic viscosity on the temperature was performed. Then the activation energy was obtained from measured values. Finally, the hysteresis areas were counted and measured values were evaluated with use of Herschel-Bulkley mathematical model.

## Introduction

The Rheological measurements of substances are very important and find applications in many fields of human activity. Determination of the rheological behaviour of substances is particularly important for designing of equipments for transport, pumping and storage of substances. Survey of the rheological behaviour also play very important role in the food rheology, where rheology among other things related with quality control and sensory properties [1]. The other applications of the rheology are for example the polymer industry [2], the building industry [3], metallurgy [4], geology and mining industry [5]. Equally important application is use of the rheology in the waste management. These include wastewater treatment, and sewage sludge utilization. Determination of rheological parameters of sewage sludge is the base for its characterization. Information about rheological properties is very important for processes, which relate with the utilization of sewage sludge. These are especially transport, dewatering, drying and landfilling of sewage sludge [6]. The rheological properties of various kinds of sewage sludge can be very different. These properties are different in various steps of wastewater treatment [7,8]. It is known that suspensions of biological sewage sludge are non-Newtonian fluids [9]. However, from the rheological point of view very thin layers behave as Newtonian fluid. When the suspension is concentrated the sludge starts to behave as non-Newtonian fluid [6]. The dependence of total solid soluble (TSS) on rheological properties of the activated sewage sludge was described by other authors [9-11].

**Figure 1:**
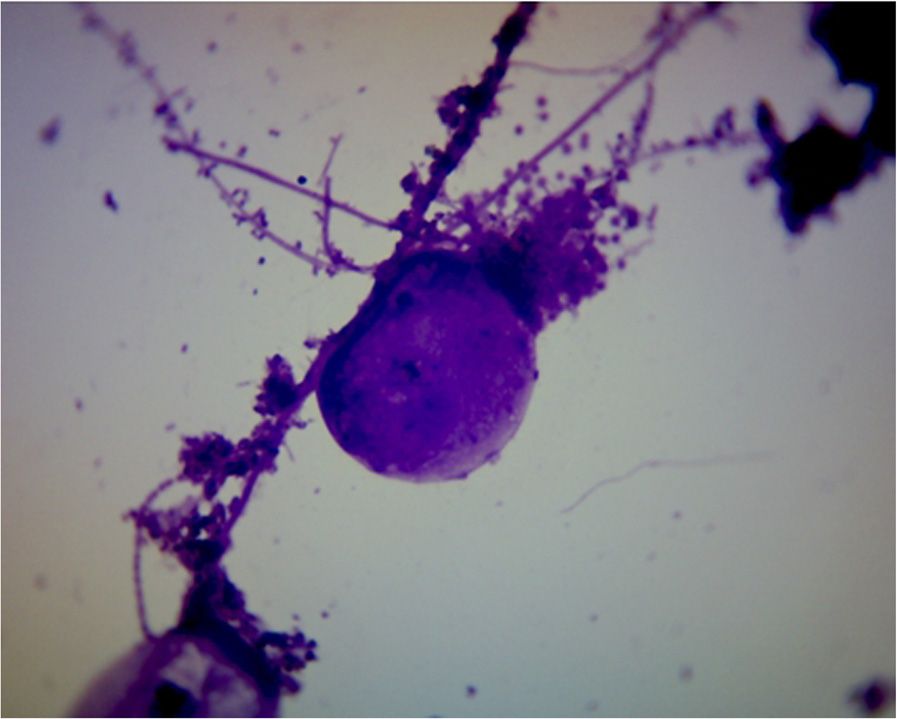
Thickened sludge structure.

**Figure 2:**
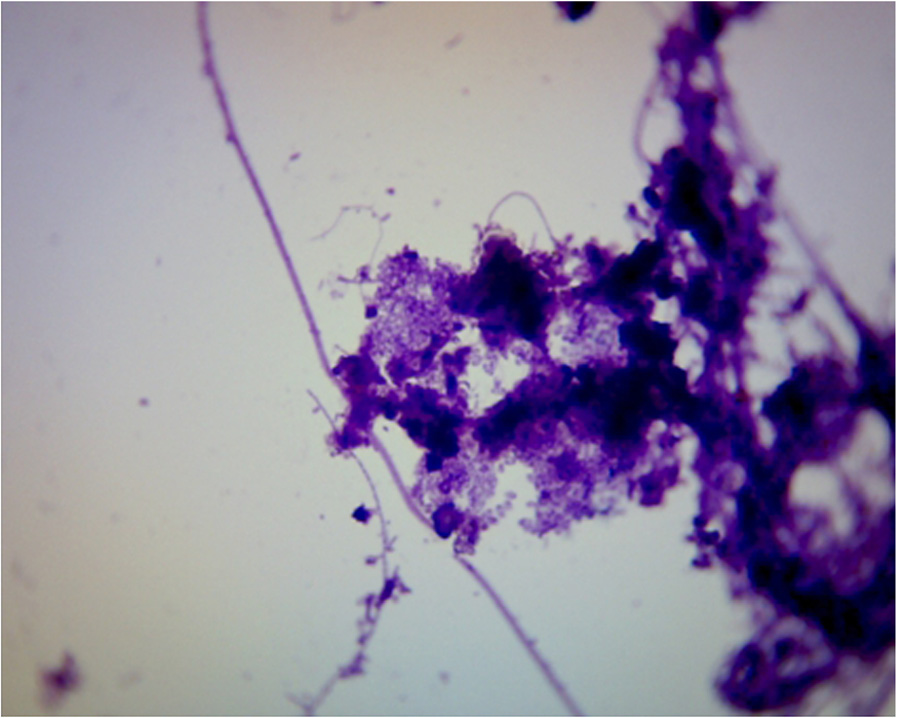
Thickened sludge structure.

This paper is focused on the rheology of thickened sludge, which was thickened by pressure flotation unit. The thickened sludge is very complicated colloidal system, which is composed of organic and inorganic particles. The organic part of thickened sludge is composed of bacteria such as bacteria of the genus *Pseudomonas*, *Flavobacterium*, *Acinetobacter*, *Achromobacte, Actinobacteria*, but also protozoa and micromycetes. The inorganic part is above all composed of the solid particles of sand. The structure of thickened sludge is showed on the Figures 1 and 2.

Problematic of pumping and transportation of thickened sludge was described for example in works of author Slater [12,13]. But rheological behaviour of thickened sludge is very little discussed in professional works. However for example Baudez et al. pointed out that rheological behaviour of activated (raw) and anaerobically digested sludge at low shear and a shear thinning at high shear stress are viscoelastic [14-16]. But these types of sludges have the thixotropic behaviour at intermediate shear stress [17]. It can be expected that the rheological behaviour of thickened sludge is very similar. As was mentioned previously the rheological behaviour of sewage sludge depends on total solid soluble, but also content organic and inorganic substances per unit volume, number of particles and particle size distribution. It is also proved that the change of a particle size distribution change also rheological properties of sewage sludge, for example after disintegration [18,19]. The goal of the paper is to extend the knowledge about rheological behaviour of thickened sewage sludge, which is not sufficiently presented in professional articles. For the reason the change of the rheological properties of thickened sludge can be observed, the researched sample was diluted in various ratios. By diluting data were obtained for various thickened sewage sludges with various dry matters.

## Material and methods

### Thickened sludge

The samples of materials were collected from flotation unit output at the wastewater treatment plant of 10 000 PE. The collection of samples was performed according to the standard ISO 10381–6: Soil quality – Sampling – Part 6. The samples were transported to the laboratory In the day of collection. The solid content in thickened sewage sludge and loss of ignition was determined according to the standard EN 12879: Characterization of sludges - Determination of the loss on ignition of dry mass. For this case the electric muffle furnace LHM 07/12 was used. The weighting of the samples was executed using of the analytical scales Radwag AS 220/X with an accuracy of 0.0001 g. The microscopy analyses of thickened sludge samples were obtained by use microscop Intraco Micro LMI B (Czech Republic). Pictures were zoomed a thousand times. For the purpose of the experiment the samples were diluted in rate 1:1, 1:2 and 1:9. Diluting was performed by drinking water, whose origin is from the same area as collected sewage sludge. The description of samples is showed in the Table 1.

**Table 1:** Description of samples

**Samples**	**Description of samples**
A	Non-diluted
B	Diluted in rate 1:1
C	Diluted in rate 1:2
D	Diluted in rate 1:9

### Measurement of rheological behaviour

There are several methods, which are designed for measurements of the rheological behaviour of substances with different types of measuring geometry such as concentric cylinders, cone and plate or parallel plates [20]. An extensive overview about measurement techniques for rheological testing is given in paper [21]. Rheological measurement of substances for this paper was performed using rheometr Anton Paar MCR 102 (Austria) with measuring geometry plate–plate. The diameter of plate was 50 mm. The constant shear test was performed with value of shear rate 50 s^-1^.

The rheological experiments were performed with non-dilute sample and with dilute samples in various rates. The flow curves were modelled by using the following model:

Herschel–Bulkley model

(1) $$\tau =\tau_{0}+K\cdot {\dot{\gamma }}^{n}$$

Where

*τ* – shear stress [Pa]

*τ*_*0*_ – yield stress [Pa]

*K* – consistency coefficient [-]

*n* – flow behaviour index [-]

$\dot{\gamma }$ – shear rate [s^-1^]

The change of dynamic viscosity in dependency on temperature was measured at temperature range 5 – 40°C. Shear rate was constant with value 50 s^-1^.

For determination of mathematical dependency between viscosity and increasing temperature was used Arrhenius mathematical model, which is given by equation:

(2) $$\eta =\eta_{0}\cdot e^{-\frac{E_{A}}{\mathrm{RT}}}\left[\mathrm{Pa}\cdot s\right]$$

Where:

*η*_*0*_ – initial value of dynamic viscosity [Pa∙s]

*E*_*A*_ – activation energy [J]

*R* – universal gas constant [J∙K^-1^∙mol^-1^]

*T* – thermodynamic temperature [K]

This model was used for an evaluation of dependence of the dynamic viscosity *η* on the temperature and for an evaluation of the activation energy *E*_*A*_. The rate of the thixtotropy was determined by equation:

All measurements were performed in three repetitions. Subsequently arithmetic mean has been evaluated from measured data. The data were tested by Grubbs test for remoteness values.

## Results and discussion

The values of basic characteristics of measured samples are showed in the Table 2. Dry matter of non-diluted sample is 3.52%. This value depends above all on selected technology of thickening. Generally sewage sludge can thicken on 3% to 6% of dry matter. Value of dry matter in other papers was similar and values are ranged 4 – 5% [22]. Values in the table also show that samples content relatively high rate of inorganic particle, approximately 35%.

**Table 2:** Base characteristics of measured samples

**Samples**	**Dry matter (%)**	**Water content (%)**	**Loss on ignition (%)**	**Residue on ignition (%)**
A	3.52	96.48	65.33	34.67
B	1.69	98.31	63.21	36.79
C	1.30	98.70	63.22	36.78
D	0.61	99.39	63.00	37.00

**Figure 3:**
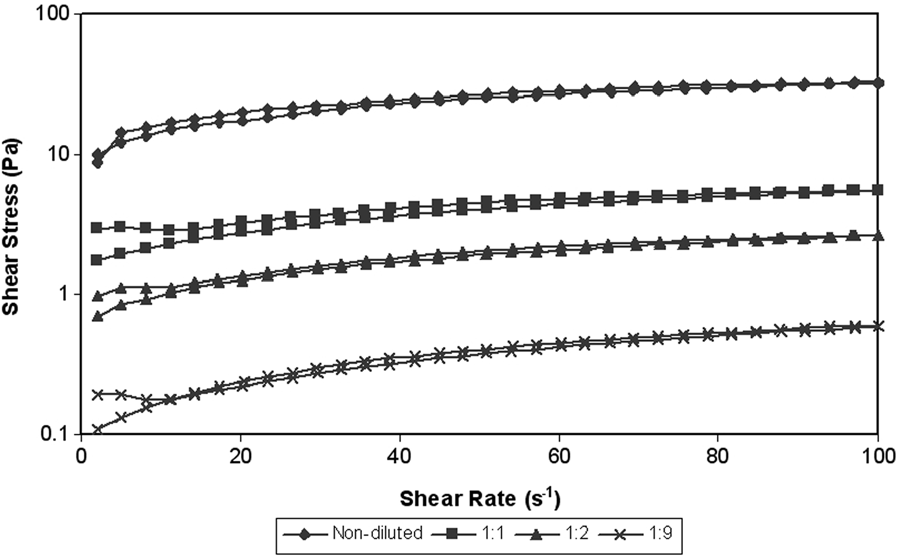
**Hysteresis loop of thickened sludge at various of dilutation rates – temperature 10°C.**

**Figure 4:**
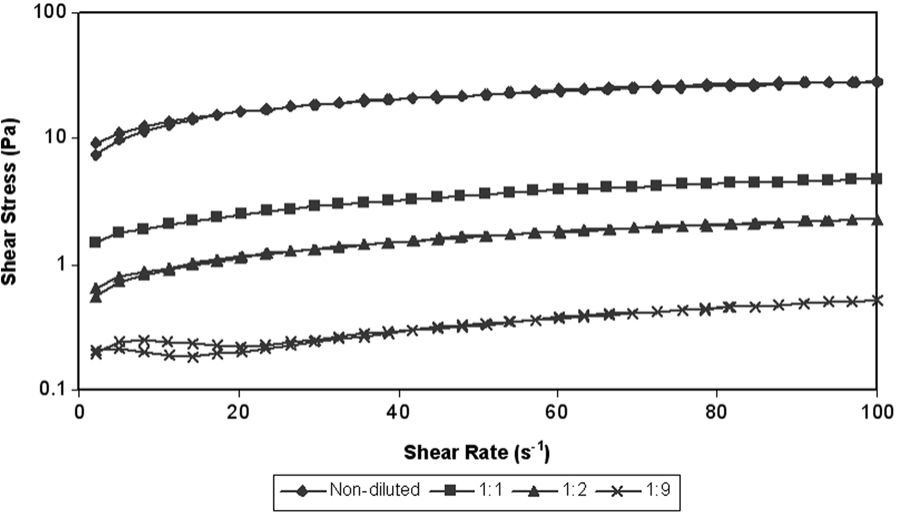
**Hysteresis loop of thickened sludge at various of dilutation rates – temperature 20°C.**

**Figure 5:**
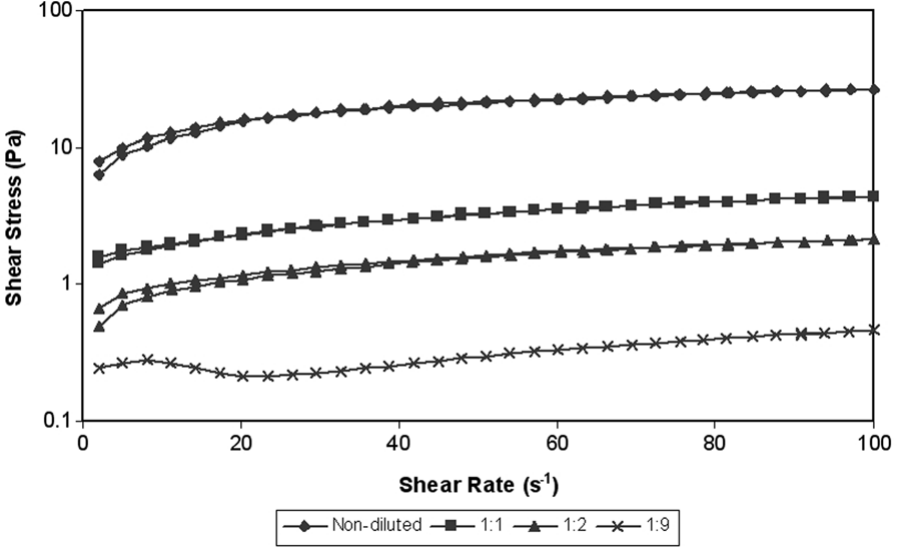
**Hysteresis loop of thickened sludge at various of dilutation rates – temperature 30°C.**

The shear rate dependences on the shear rate at various temperatures (10°C, 20°C, 30°C) are showed in Figures 3, 4 and 5. During the test the shear stress increase gradually to the maximal value 32 Pa (sample A at temperature 10°C). During decreasing of the shear rate the measured values of shear stress started to generate hysteresis loop. The hysteresis loop was generated at all measured samples. Subsequently hysteresis area was counted from hysteresis loop. The counted values are given in the Table 3. Hysteresis area values of measured samples were relatively low, but the measured values are in the accordance with other works, where rheological properties of activated sludge were measured [23]. However according to Guibaud et al. [23] the values of hysteresis area increase with the development of filamentous in the sewage sludge. The value of hysteresis area can be considered as a measure of the degree of thixotropy [24]. However the hysteresis area cannot be a sole measure for evaluating of thixotropy or antithixotropy. For example the author Baudez et al. [25] presents that hysteresis area is simply a consequence of the shear localization rather than a thixotropic behaviour and its area is closely linked to the apparatus and the data sampling [25]. Thixotropy is based on three essential elements: it is based on viscosity; it impiles a time-dependent decrease of the viscosity induced by flow; the effect is reversible when the flow is decreased or arrested [26]. This means that the loop test is only approximate test for rheology evaluating of samples.

**Table 3:** Hysteresis area

**Samples**	**Hysteresis area (Pa · s**^**-1**^**)**		
	**10°C**	**20°C**	**30°C**
A	- 1323.05	- 1272.96	- 1211.56
B	- 205.06	- 203.23	- 194
C	- 104.21	- 97.61	- 94.62
D	- 22.93	- 20.04	- 17.96

**Figure 6:**
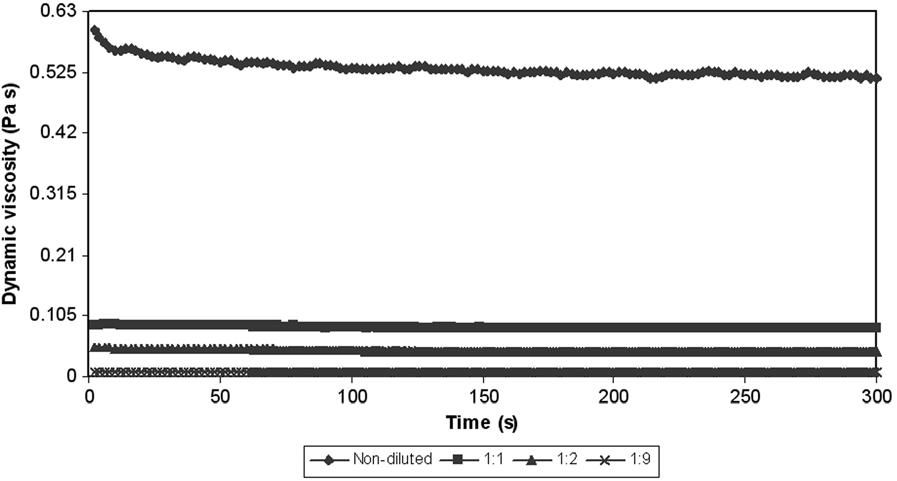
**Dependence of dynamic viscosity on the time at constant shear rate (50 s**^**-1**^**) and at the constant temperature (10°C).**

**Figure 7:**
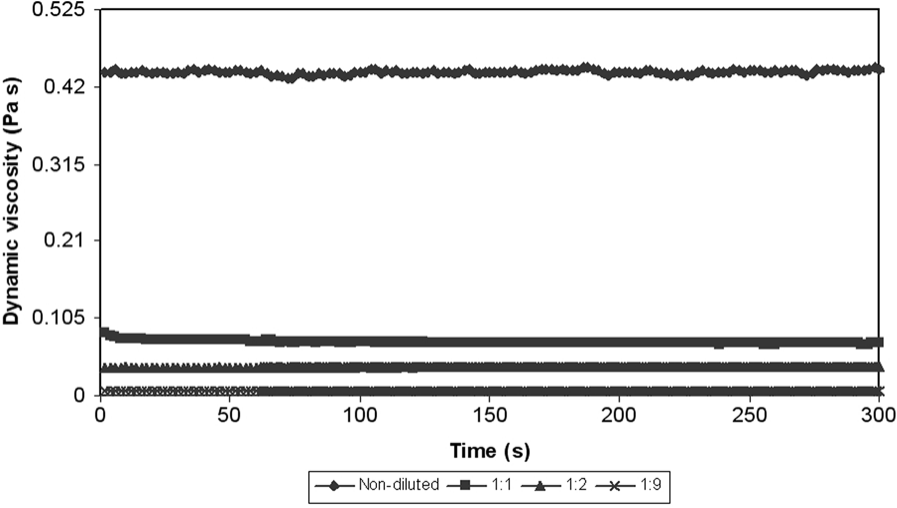
**Dependence of dynamic viscosity on the time at constant shear rate (50 s**^**-1**^**) and at the constant temperature (20°C).**

**Figure 8:**
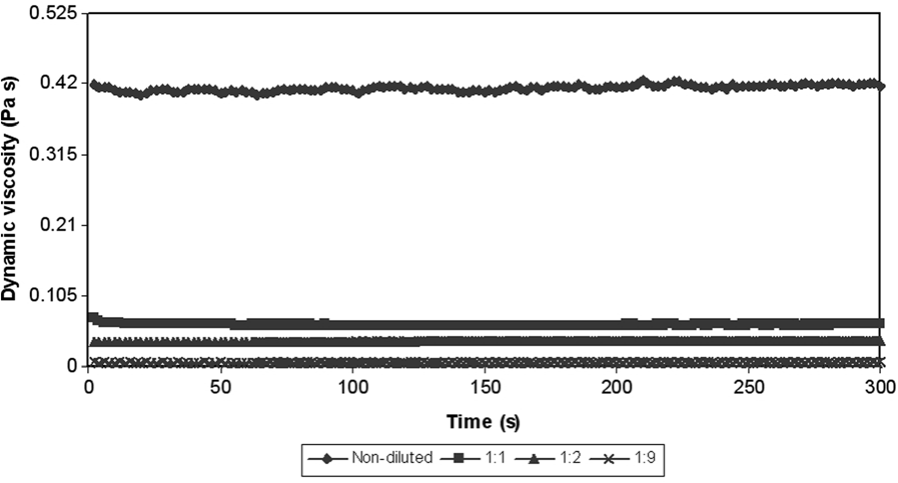
**Dependence of dynamic viscosity on the time at constant shear rate (50 s**^**-1**^**) and at the constant temperature (30°C).**

From the reason the next tests were performed. The next test was related to the measurement of dependence of dynamic viscosity on the time at constant shear rate (50 s^-1^). Results of these tests for various temperatures are showed on the Figure 6, 7 and 8. In all cases there was a decrease of a viscosity in the time. In other words the measured samples are time-dependent. There are two posibilities, which can occur. The measured sample is thixotropic or viscoelastic material. Because both the thixotropy and the viscoelasticity express time and shear history effects [26]. But in accordance with the above-mentioned the measured samples can be with the highest probability considered for substances with thixotropic behaviour.

Classical rheological models used for non-Newtonian fluids such as Bingham’s or Herschel-Bulkley’s can be used for description of rheological behaviour of sewage sludges [27]. For the purpose of the paper Herschel-Bulkley mathematical model was chosen. Results of Herschel-Bulkley’s models evaluation are showed in the Table 4.

**Table 4:** Evaluating Herschel-Bulkley model for non-diluted sewage sludge

**Samples**	**Temperature (°C)**	**R**^**2**^** (-)**	Standard dev.(Pa)*	**Yield stress (Pa)**	**Consistency (Pa)**	**Flow index (-)**
A	10	0.98002	0.80686	3.1671	4.8647	0.38979
	20	0.99519	0.35269	2.4858	4.1851	0.39726
	30	0.9945	0.3567	**	5.4786	0.34439
B	10	0.93507	0.25183	1.7391	0.15084	0.70647
	20	0.99693	0.049395	1.2548	0.16706	0.66263
	30	0.99806	0.035527	1.2437	0.14173	0.67471
C	10	0.98335	0.066535	0.65045	0.08747	0.68346
	20	0.99873	0.01605	0.42062	0.12119	0.59243
	30	0.99184	0.036811	0.32042	0.18062	0.4988
D	10	0.98472	0.016815	0.11033	0.00887	0.87658
	20	0.97756	0.015108	0.19508	0.00048	1.4314
	30	0.94222	0.019159	0.21201	0.00005	1.8729

*relating to yield stress.

**makes no sense rheologically.

Measured values of dynamic viscosities of all samples were subjected to further mathematical analysis. Dynamic viscosities were determined from the time-dependence test. Arrhenius mathematical model was used for this analysis. This model is showed in equation (2). The logarithm of this equation is:

(3) $$\ln\eta =\ln\eta_{0}+\frac{E_{A}}{R\cdot T}$$

The activation energy *E*_*A*_ was performed by using of this equation with use of regression analysis. Values of activation energies are showed in the Table 5.

**Table 5:** Activation energy of individual samples

**Samples**	**R**^**2**^** (-)**	**Activation energy(J · mol**^**-1**^**)**
A	0.9498	8871
B	0.9876	9411
C	0.9888	8389
D	0.9949	11556

The table shows that activation energy is dependence on the dry matter of the sample. It means that in case of decreasing of dry matter values of activation energy increases. For comparison for example Yang et al. presents that the activation energy of activated sewage sludge from membrane technology was 9217 J∙mol^-1^[28] and activation energy of activated sewage sludge was 6000 J∙mol^-1^[29]. The values are thus very similar.

## Conclusion

The rheological measurements were performed by rotational rheometer with geometry plate–plate. It has been showed that thickened sewage sludge has the similar rheological properties as activated sewage sludge or digested sewage sludge. Similarly as these types of sludges the thickened sewage sludge has thixotropic behaviour in intermediate shear stress. The rate of a thixotropy decreases with an increase of water content in the sample. It was also showed that Herschel-Bulkley mathematical model is very suitable for evaluating of shear stress dependence on the shear rate too. Determination coefficient was ranged mostly about 0.99. The calculation of activation energy was performed by using Arrhenius mathematical model. The values of activation energy were very similar as other types of sewage sludges, such as activated sludges.

## Competing interests

The authors declare that they have no competing interests.

## Authors’ contribution

PT: measuring of data, data processing, evaluation of data, writing of paper. PJ: collection of samples preparation of samples, preparation of tables and graphs. Both authors read and approved the final manuscript.
